# A Novel Epithelial-Mesenchymal Transition Gene Signature Correlated With Prognosis, and Immune Infiltration in Hepatocellular Carcinoma

**DOI:** 10.3389/fphar.2022.863750

**Published:** 2022-04-20

**Authors:** Weihao Kong, Zhongxiang Mao, Chen Han, Zhenxing Ding, Qianqian Yuan, Gaosong Zhang, Chong Li, Xuesheng Wu, Jia Chen, Manyu Guo, Shaocheng Hong, Feng Yu, Rongqiang Liu, Xingyu Wang, Jianlin Zhang

**Affiliations:** ^1^ Department of Emergency Surgery, The First Affiliated Hospital of Anhui Medical University, Hefei, China; ^2^ Registration Review Department, Anhui Center for Drug Evaluation & Inspection, Hefei, China; ^3^ Department of Emergency Medicine, The First Affiliated Hospital of Anhui Medical University, Hefei, China; ^4^ Department of Biochemistry & Molecular Biology, School of Basic Medicine, Anhui Medical University, Hefei, China; ^5^ Department Ultrasound, The First Affiliated Hospital of Anhui Medical University, Hefei, China; ^6^ The First Clinical Medical College of Anhui Medical University, Anhui Medical University, Hefei, China; ^7^ Department of Hepatobiliary Surgery, The First Affiliated Hospital of Guangzhou Medical University, Guangzhou, China

**Keywords:** epithelial-mesenchymal transition, gene signature, TCGA, GEO, ICGC, hepatocellular carcinoma

## Abstract

**Background:** Although many genes related to epithelial-mesenchymal transition (EMT) have been explored in hepatocellular carcinoma (HCC), their prognostic significance still needs further analysis.

**Methods:** Differentially expressed EMT-related genes were obtained through the integrated analysis of 4 Gene expression omnibus (GEO) datasets. The univariate Cox regression and Lasso Cox regression models are utilized to determine the EMT-related gene signature. Based on the results of multivariate Cox regression, a predictive nomogram is established. Time-dependent ROC curve and calibration curve are used to show the distinguishing ability and consistency of the nomogram. Finally, we explored the correlation between EMT risk score and immune immunity.

**Results:** We identified a nine EMT-related gene signature to predict the survival outcome of HCC patients. Based on the EMT risk score’s median, HCC patients in each dataset were divided into high and low-risk groups. The survival outcomes of HCC patients in the high-risk group were significantly worse than those in the low-risk group. The prediction nomogram based on the EMT risk score has better distinguishing ability and consistency. High EMT risk score was related to immune infiltration.

**Conclusion:** The nomogram based on the EMT risk score can reliably predict the survival outcome of HCC patients, thereby providing benefits for medical decisions.

## Introduction

Liver cancer is a common malignant tumor that has become a danger to public health globally. Hepatocellular carcinoma is well-established as the predominant pathological type and is mainly caused by hepatitis virus infection, alcoholism, and liver cirrhosis (McGlynn et al., 2020; Li et al., 2021; Siegel et al., 2021). Current evidence suggests that HCC patients with the same pathological type may have different survival outcomes, suggestive of the molecular heterogeneity in the same tumor type, accounting for the difficulty in predicting the survival outcome of HCC patients (El-Serag and Kanwal, 2014; Caruso et al., 2020). Therefore, understanding the molecular changes in HCC patients may provide new treatment strategies and improve patient prognosis.

Epithelial-mesenchymal transition (EMT) refers to the transformation process of cells from epithelial cells to mesenchymal cells, crucial in wound healing, tumor progression, and embryonic development (Ribatti et al., 2020). Its main functions involve the reduction of cell adhesion molecule (such as E-cadherin) expression and converting cytokeratin cytoskeleton into a vimentin-based cytoskeleton that exhibits the morphological characteristics of mesenchymal cells. During the process of EMT, epithelial cells lose cell polarity and the ability to adhere to the basement membrane, thus acquiring phenotypes such as invasion, migration, chemoresistance, and anti-apoptosis. An increasing body of evidence suggests that EMT is an essential step in initiating tumor development, suggesting its potential as a therapeutic target to prevent HCC progression (Zheng et al., 2015; Dominguez et al., 2017; Shibue and Weinberg, 2017; Ribatti et al., 2020).

Given the close association between EMT and the occurrence and development of HCC, the present research sought to systematically evaluate the prognostic role of EMT-related genes. Four HCC microarrays from the GEO dataset were integrated, and differentially expressed EMT-related genes were analyzed. A prognostic EMT-related gene signature was established through the univariate and LASSO Cox regression model, and a nomogram model was constructed based on the EMT risk score. Finally, we explored the correlation between EMT risk score and tumor immunity.

## Materials and Methods

### Data Acquisition and Processing

We downloaded the raw data from TCGA (https://portal.gdc.cancer.gov/), ICGC (https://icgc.org/), and GEO (https://www.ncbi.nlm.nih.gov/geo/) website, processed the raw data, and finally obtained the expression matrix of hepatocellular carcinoma tissues (HCC). Datasets GSE22058, GSE25097, GSE36376, and GSE39791 containing data on HCC and para-cancerous tissues were used to screen differentially expressed genes (DEGs). All datasets were normalized by Robust Multi-Array Average (RMA), and different probes were converted into gene symbols. The dbEMT2 database (http://dbemt.bioinfo-minzhao.org/download.cgi) was used to download EMT-related genes. Perl language was used to extract the expression matrix of EMT-related genes from the TCGA, ICGC, and GSE14520 HCC datasets in batches.

### Integrated Analysis of Epithelial-Mesenchymal Transition-Related Gene Datasets

The MetaDE package was used to screen DEGs between HCC and para-cancerous tissues (Ramasamy et al., 2008; Wang et al., 2012). Before performing the microarray meta-analysis, we extracted common genes between different datasets and eliminated the top 30% genes with average signal intensity and standard deviation. The four methods Fisher, maxP, roP, and AW were used to analyze differentially expressed genes (DEGs). A corrected *p*-value of less than 0.05 was statistically significant. Given the heterogeneity among different statistical methods, the R package VennDiagram was used to identify common differentially expressed EMT-correlated genes.

### Functional Enrichment Analysis

The clusterProfiler package was used to identify the biological functions and pathways of differentially expressed EMT-related genes (Yu et al., 2012). Gene Ontology (GO) annotation was conducted for three domains: cellular component (CC), molecular function (MF), and biological process (BP). A bubble plot was used to visually display the results of GO and KEGG function analyses. Gene set enrichment analysis (GSEA) was used to evaluate the distribution of genes in a predefined gene set. Results were displayed in a gene table with genes ranked by their relevance to phenotype, reflecting their contribution to the phenotype. The input data consisted of two parts, a gene set with known functions and an expression profile matrix, to explore significantly enriched pathways between the high and the low EMT risk score group. An FDR and *p*-value of less than 0.05 were statistically significant.

### Identification of a Prognostic Epithelial-Mesenchymal Transition-Correlated Gene Signature

After excluding HCC patients who survived less than 30 days, 342, 221, and 230 patients remained in the TCGA, GSE14520, and ICGC datasets, respectively. The prognostic-related EMT genes were determined in the TCGA and GSE14520 datasets, yielding 43 common EMT-related prognostic genes based on the univariate Cox regression model. Then, we used the least absolute shrinkage and selection operator (LASSO) cox regression model to further screen the EMT-related risk genes in the TCGA dataset and finally determined 9 EMT gene signatures could predict hepatocellular carcinoma prognosis. The EMT risk score is equal to the sum of the product of EMT gene expression and its coefficient. We calculated the EMT risk score of each patient in the TCGA, GSE14520, and ICGC datasets and divided HCC patients into a high and a low EMT risk score group based on the median value in each dataset. The TCGA dataset was used to construct the EMT gene signature, while the GSE14520 and ICGC datasets were used to validate the constructed signature. The survival curve between high and low-risk groups of HCC patients was generated using the survminer package, and the difference in survival times was assessed by the log-rank test. The survival ROC package assessed the predictive performance of the EMT risk score for survival outcomes.

### Nomogram Construction and Validation

The nomogram was based on multivariate regression analysis, which integrates multiple predictors, and scaled line segments were drawn on the same plane according to a certain proportion to ensure that the survival probability of patients can be predicted objectively. In the TCGA dataset, we included patients with exact age, sex, tumor differentiation, tumor stage, and survival information. Univariate and multivariate cox regression models were used to screen variables that affect survival outcomes (including OS and RFS). Therefore, the risk score and tumor stage data from the TCGA dataset were included to build a predictive nomogram model for OS and RFS. Datasets GSE14520 and ICGC were used for external validation. The time-dependent ROC curve was used to assess the predictive ability, and the consistency of the nomogram model was visually represented by the 1-year, 3-year, and 5-year calibration curve. The larger the area under the time-dependent ROC curve, the better the discriminative ability of the nomogram model. The higher the degree of coincidence between the actual 1-year, 3-year, and 5-year calibration curves and the lines at the 45-degree angle, the better the consistency.

### Human Protein Atlas Validation

The Human Protein Atlas (http://www.proteinatlas.org/) database is a well-recognized online database that provides tissue and cell distribution information of all 24,000 human proteins. It can provide the expression of a specific protein in tumor tissues and normal tissues. In the present study, we analyzed differences in protein expression of these 9 EMT risk genes between HCC and normal liver tissues.

### Epithelial-Mesenchymal Transition-Related Gene Signature Correlated With Immune Infiltration

The TIMER (Tumor Immune Estimation Resource) database (https://cistrome.shinyapps.io/timer/) is an online database that provides information on the infiltration status of the six immune cells (B cells, CD4^+^ T cells, CD8^+^ T cells, Neutrophils, Macrophages, and Dendritic cells) in the TCGA database (Li et al., 2017). Besides, we downloaded the immune score, stromal score, and ESTIMATE score of HCC tissues of the TCGA dataset from the ESTIMATE database (https://bioinformatics.mdanderson.org/estimate). Correlation analysis was used to explore the correlation between EMT risk score and immune-related indexes (including six immune cell types, immune score, stromal score, and ESTIMATE score). Immune checkpoint genes included CD274 (PD-L1), PDCD1(PD-1), TIGIT, CTLA4, HAVCR2, and ICOS. Finally, we analyzed the differences in mRNA expression levels of immune checkpoint genes between high and low-risk groups.

### Statistical Analysis

All statistical analyses were conducted using R3.6.1 and SPSS26.0 software. Chi-square analysis was used to analyze the correlation between the EMT risk score group and clinicopathological characteristics of HCC patients. EMT genes with a *p*-value less than 0.05 during univariate analysis were included in the LASSO Cox regression model. STATA software was used to explore the combined effect between EMT risk score and survival outcome. A *p*-value less than 0.05 was statistically significant.

## Results

### Identification of Dysregulated Epithelial-Mesenchymal Transition-Related Genes

**FIGURE 1:**
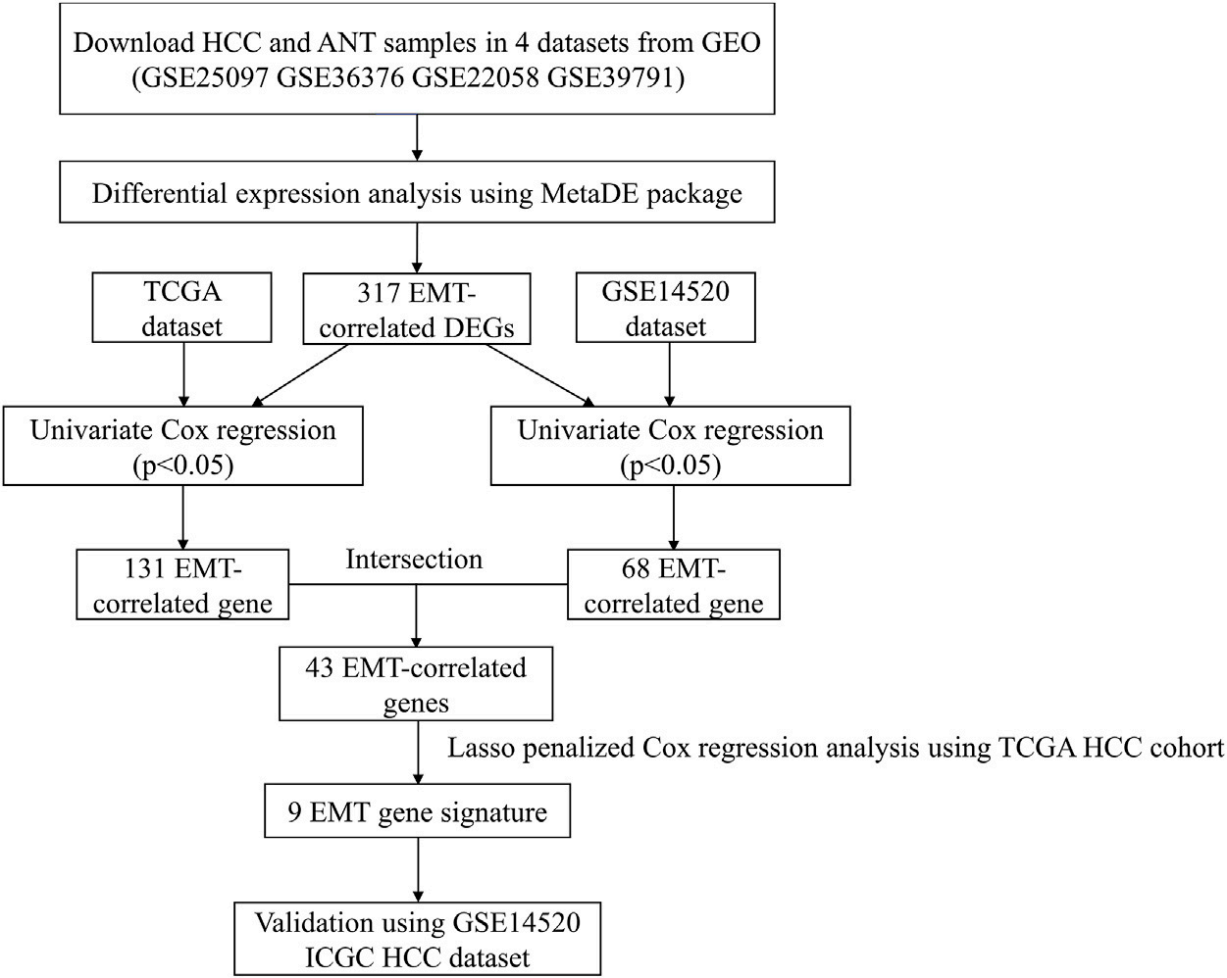
The workflow of this study.

**FIGURE 2:**
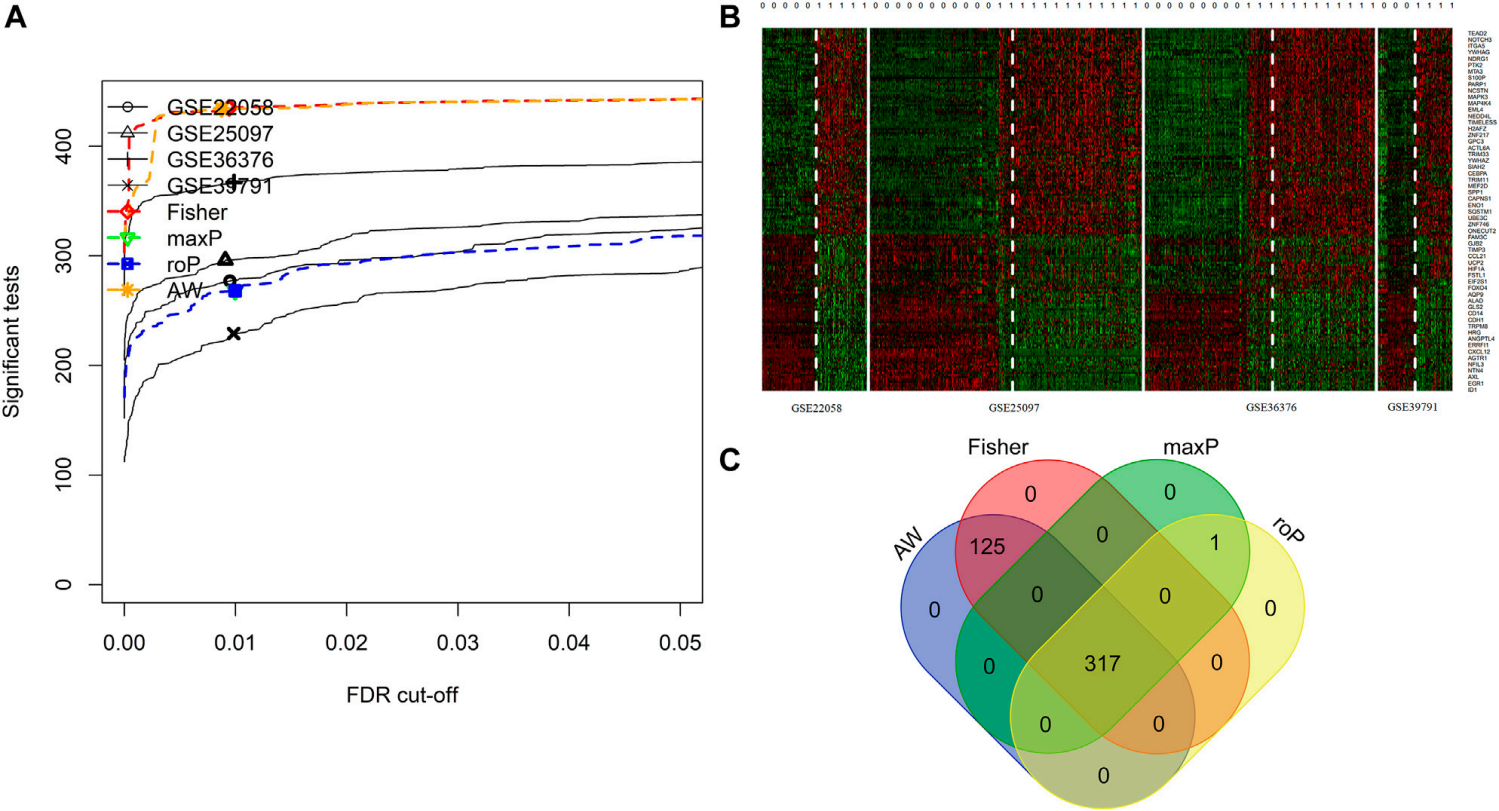
Integrated analysis of differentially expressed EMT-correlated genes. **(A)**. Four different meta-analysis algorithms (Fisher, roP, maxP, AW) were used to identify differentially expressed genes in four HCC datasets. **(B)** Heatmap of EMT correlated-genes (FDR<1E-19) in four HCC cohorts. **(C)** Veen diagram shows the differential expressed genes obtained by the four algorithms. Notes: HCC, hepatocellular carcinoma; EMT, Epithelial-mesenchymal transition.

4 HCC cohorts containing HCC and para-cancerous tissues were integrated. The details of the four cohorts are shown in Table 1. The detailed process of this research is displayed in Figure 1. The AW, Fisher, maxP, and roP methods yielded 442, 442, 318, and 318 EMT-related differentially expressed genes, respectively (Figure 2A). Figure 2B shows the expression matrix heat map of the EMT related-genes with FDR values less than 1E-19 in the four HCC cohorts using the maxP method. Finally, we identified 317 differentially common expressed EMT-related genes (Figure 2C).

**TABLE 1:** Basic characteristics of the included HCC dataset.

Author	Publication year	Country	Manufacturer	Number of probes	Size (Non-tumor/Tumor)	Platform	Source accession
Chunsheng Zhang	2011	United States	Affymetrix	37,582	511 (243/268)	GPL10687	GSE25097
Cheol-Keun Park	2012	Korea	Illumina Inc.	48,107	433 (193/240)	GPL10558	GSE36376
Julja Burchard	2010	United States	Affymetrix	43,483	197 (97/100)	GPL6793	GSE22058
Sangbae Kim	2014	United States	Illumina Inc.	48,107	144 (72/72)	GPL10558	GSE39791

HCC, hepatocellular carcinoma.

### Functional Enrichment Analysis

**FIGURE 3:**
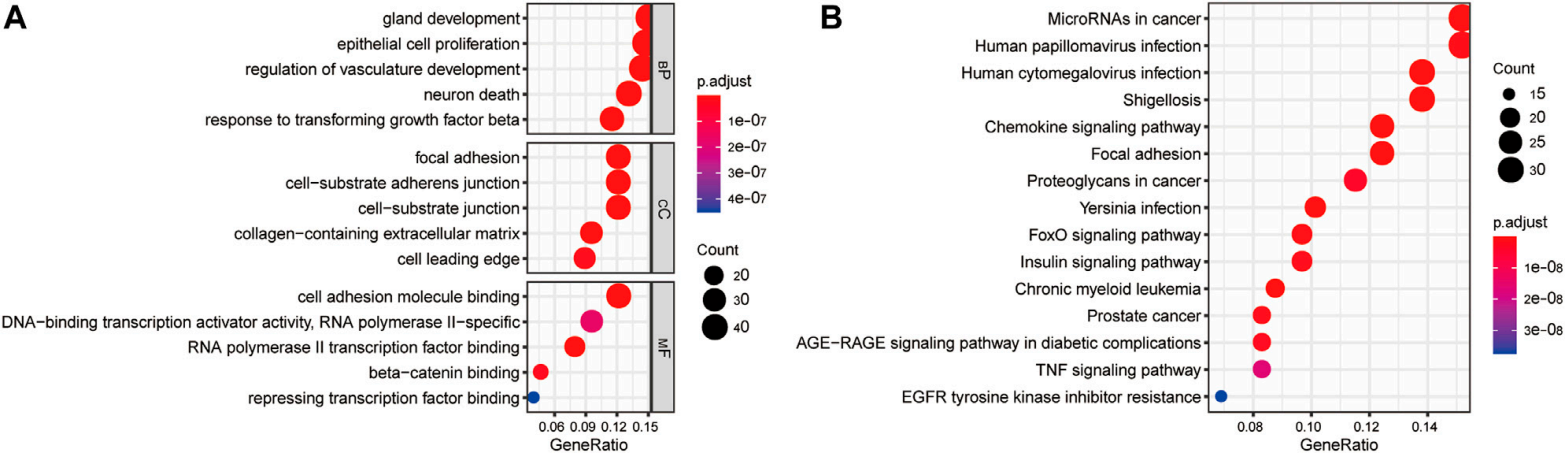
Gene enrichment analysis of differentially expressed EMT-correlated genes. **(A)**. Enriched GO terms of differentially expressed EMT-correlated genes (Top5). **(B)** Enriched pathways of differentially expressed EMT-correlated genes (Top15). Notes: EMT, Epithelial-mesenchymal transition; GO, gene ontology; BP, Biological Process; CC, Cellular Component; MF, Molecular Function.

The clusterProfiler package was used to explore the functions and pathways of differentially expressed EMT-related genes. The top 5 significantly enriched GO terms in the BP domain included gland development, epithelial cell proliferation, regulation of vasculature development, neuron death, and response to transforming growth factor-beta (Figure 3A). The top 5 significant GO CC terms included focal adhesion, cell-substrate adherens junction, cell-substrate junction, collagen-containing extracellular matrix, and cell leading edge (Figure 3A). Moreover, the top 5 significantly enriched MF GO terms were RNA polymerase II transcription factor binding, cell adhesion molecule binding, beta-catenin binding, DNA-binding transcription activator activity, and repressing transcription factor binding (Figure 3A). Finally, the differentially expressed EMT-related genes were significantly enriched in KEGG pathways, including MicroRNAs in cancer, Human papillomavirus infection, Human cytomegalovirus infection, Shigellosis, and Chronic myeloid leukemia (top 5) (Figure 3B).

### Construction and Validation of an Epithelial-Mesenchymal Transition-Related Gene Signature

**FIGURE 4:**
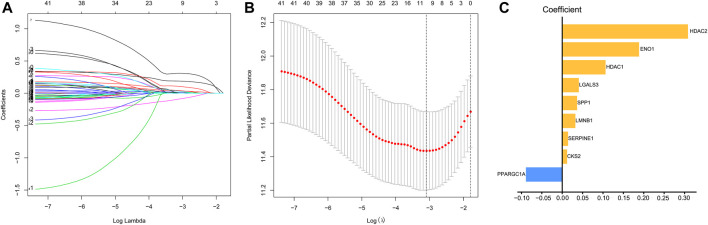
Identification of EMT risk genes that affect OS in HCC patients in the TCGA dataset. **(A, B)**. The lasso-Cox regression model was utilized to identify EMT-related gene signature through 10-fold cross-validation. **(C)** The coefficient of the selected EMT risk genes. Notes: EMT, Epithelial-mesenchymal transition; OS, overall survival; TCGA, The Cancer Genome Atlas Program.

**FIGURE 5:**
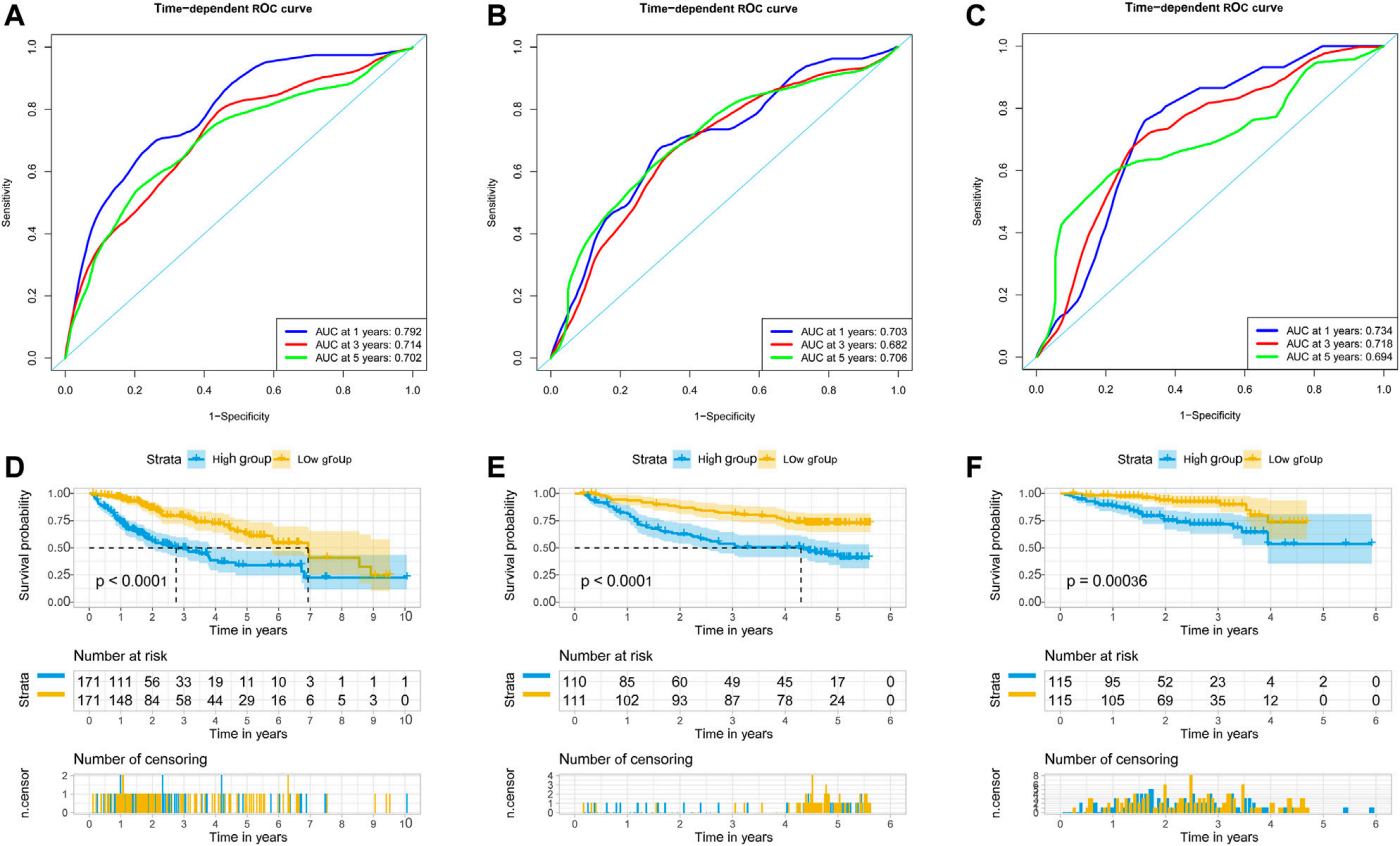
EMT-correlated gene signature associated with HCC patient’s overall survival. **(A)**. The predictive value of EMT risk score for predicting the OS of HCC patients in TCGA dataset. **(B)**. The predictive value of EMT risk score for predicting the OS of HCC patients in the GSE14520 dataset. **(C)**. The predictive value of EMT risk score for predicting the OS of HCC patients in ICGC dataset. **(D)**. The overall survival between the high EMT risk score group and the low EMT risk score group in the TCGA HCC cohort. **(E)**. The overall survival between the high EMT risk score and the low EMT risk score group in the GSE14520 HCC cohort. **(F)**. The overall survival between the high EMT risk score and the low EMT risk score group in the ICGC HCC cohort. Notes: HCC, hepatocellular carcinoma; EMT, Epithelial-mesenchymal transition; OS, overall survival; TCGA, The Cancer Genome Atlas Program; ICGC, International Cancer Genome Consortium.

**FIGURE 6:**
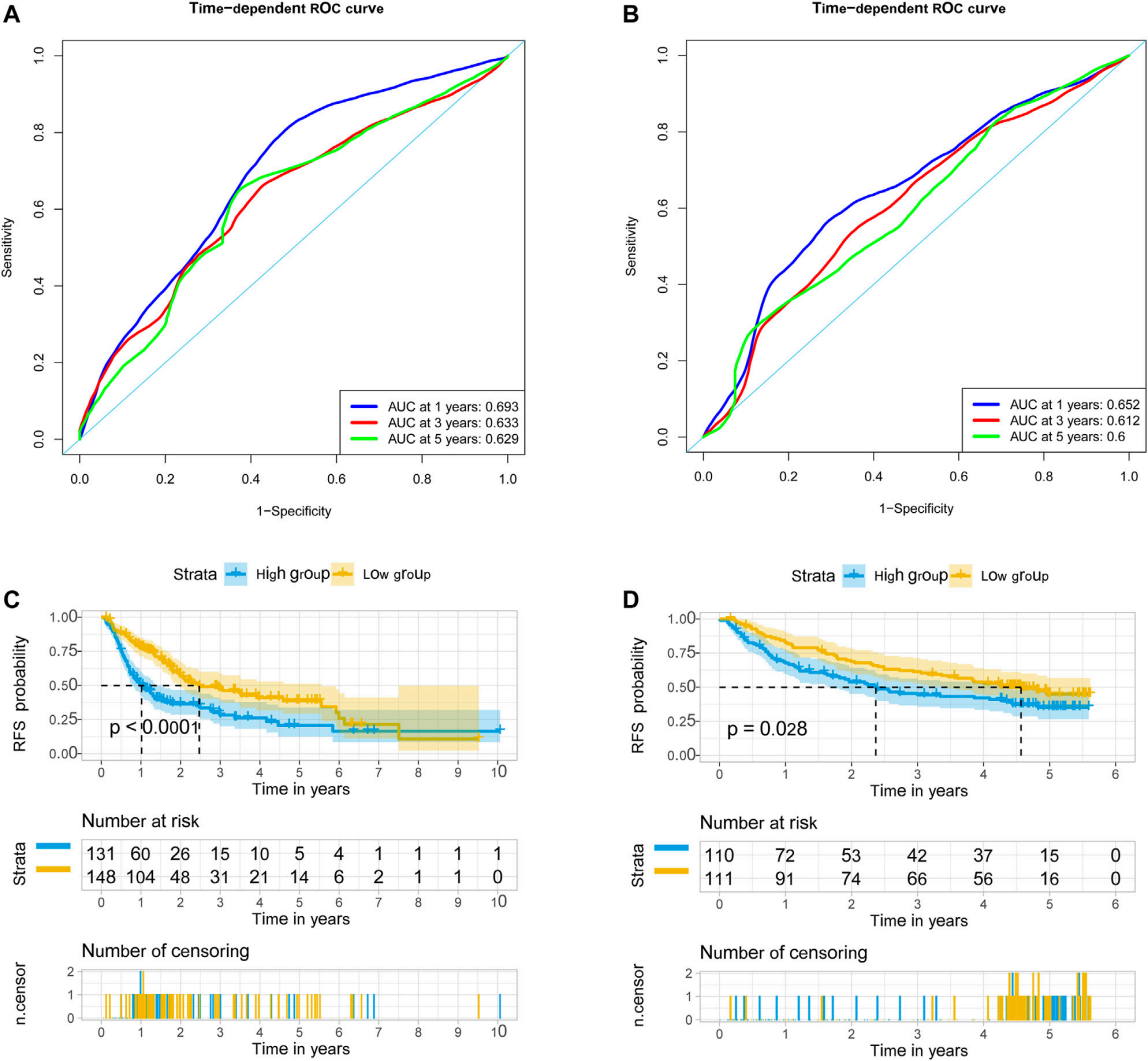
EMT-correlated gene signature associated with HCC patient’s recurrence-free survival. **(A)**. The predictive value of EMT risk score for predicting the RFS of HCC patients in TCGA dataset. **(B)** The predictive value of EMT risk score for predicting the RFS of HCC patients in the GSE14520 dataset. **(C)** The recurrence-free survival between the high EMT risk score group and the low EMT risk score group in the TCGA HCC cohort. **(D)**. The recurrence-free survival between the high EMT risk score group and the low EMT risk score group in the GSE14520 HCC cohort. Notes: EMT, Epithelial-mesenchymal transition; HCC, hepatocellular carcinoma; RFS, recurrence-free survival; TCGA, The Cancer Genome Atlas Program.

We used univariate Cox regression models to analyze the correlation between EMT-related genes and overall survival in the TCGA and GSE14520 datasets to establish a robust EMT-related gene signature. In the TCGA and GSE14520 datasets, we identified 131 and 68 EMT prognostic-related genes, respectively, and finally got 43 common prognostic EMT-related genes. Based on the TCGA dataset, the Lasso-Cox regression model identified a 9 EMT-related gene signature through 10-fold cross-validation (Figures 4A,B). The coefficients of the screened EMT genes are shown in Figure 4C. The formula for calculating the EMT risk score is as follows: 0.3084* HDAC2 + 0.0364* SPP1−0.0890* PPARGC1A+ 0.0407* LGALS3 + 0.1885* ENO1+ 0.0322* LMNB1+ 0.0119* CKS2+ 0.0143* SERPINE1+ 0.1062* HDAC1. According to the median EMT risk scores of HCC patients in the TCGA, GSE14520, and ICGC datasets, we divided HCC patients into low-risk and high-risk groups. In the TCGA dataset, the AUC values of the EMT risk score for predicting the overall survival of HCC patients at 1, 3, and 5 years were 0.792, 0.714, and 0.702, respectively (Figure 5A). HCC patients in the low EMT risk score group exhibited a better overall survival rate than HCC patients in the high EMT risk score group (*p* < 0.001) (Figure 5D). Similarly, in the GSE14520 dataset, the predictive performance of the EMT risk score for the overall survival of HCC patients at 1, 3, and 5 years was 0.703, 0.682, and 0.706 (Figure 5B). HCC patients with low EMT risk scores were associated with a longer overall survival time than HCC patients with high EMT risk scores (*p* < 0.001) (Figure 5E). In the ICGC validation set, the EMT risk score exhibited a better predictive power for overall survival (Figure 5C), and HCC patients with a high EMT risk score were associated with worse overall survival than patients with low EMT risk scores (Figure 5F). Finally, we explored the predictive power of the EMT risk score in the TCGA dataset and the GSE14520 dataset for HCC recurrence. The results of the time-dependent ROC curve showed that the EMT risk score has a moderate diagnostic value in the TCGA and GSE14520 datasets for predicting RFS (Figures 6A,B). The survival analysis results showed that HCC patients with high EMT risk scores had shorter recurrence-free time than HCC patients with low EMT risk scores (Figures 6C,D). Supplementary Figure S1 shows the distribution of EMT risk scores (A, B, C), patient survival status (D, E, F), and the expression heat map of the selected EMT genes (G, H, I) in the TCGA, GSE14520, and ICGC datasets.

### Correlation Analysis of Epithelial-Mesenchymal Transition Risk Score and Clinicopathological Characteristics

Clinicopathological characteristics associated with the TCGA, GSE14520, and ICGC datasets, including age, gender, ALT, history of malignancy, Histopathological grade, tumor stage, and survival status, were extracted. In the TCGA dataset, a significant correlation was found between the EMT risk score and histopathological grade (*p* < 0.001), tumor stage (*p* < 0.001), and survival status (*p* < 0.001). In the GSE14520 dataset, the EMT risk score was significantly correlated to age (*p* = 0.043), TNM stage (*p* < 0.001), and survival status (*p* < 0.001). In the ICGC dataset, the EMT risk score was significantly correlated to the TNM stage (*p* < 0.001) and survival status (*p* = 0.001) (Table 2).

**TABLE 2:** Correlation between EMT risk score and clinicopathological characteristics in HCC dataset.

Variables	TCGA (n = 321)			GSE14520 (n = 219)			ICGC (n = 230)		
	Low risk	High risk	*p*-Value	Low risk	High risk	*p*-Value	Low risk	High risk	*p*-Value
Age (years)			0.538			0.043			0.868
<60	77	83		83	94		22	23	
60	82	77		27	15		93	92	
Gender			0.354			0.448			0.455
male	113	106		93	96		33	28	
female	46	54		17	13		82	87	
ALT						0.308			
50	-	-		69	61		-	-	
>50	-	-		41	48		-	-	
Prior-Malignancy									0.240
no	-	-		-	-		103	97	
yes	-	-		-	-		12	18	
Histologic grade			**<0.001**						
G1/2	114	84		-	-		-	-	
G3/4	45	76		-	-		-	-	
TNM stage			**<0.001**			**<0.001**			**<0.001**
I/II	134	102		97	73		85	57	
III/IV	25	58		13	36		30	58	
Survival status			**<0.001**			**<0.001**			**0.001**
alive	124	88		82	53		104	85	
dead	35	72		28	56		11	30	

EMT, Epithelial-mesenchymal transition; HCC, hepatocellular carcinoma; ALT, alanine transaminase; TNM, tumor node metastases; TCGA, the cancer genome atlas; ICGC, international cancer genome consortium.

Bold value represent *p* < 0.05.

### Identification of Independent Variables Correlated With Survival Outcome in Hepatocellular Carcinom Cohort

In the TCGA dataset, the EMT risk score (OS: *p* < 0.001; RFS: *p* = 0.005) and tumor stage (OS: *p* < 0.001; RFS: *p* < 0.001) were independent risk factors that affected the overall survival and recurrence-free survival of HCC patients (Supplementary Table S1, S2). In the GSE14520 dataset, the EMT risk score (*p* < 0.001) and tumor stage (*p* < 0.001) were independent risk factors that affected the overall survival of HCC patients (Supplementary Table S3). Moreover, the tumor stage (*p* = 0.001) was an independent predictor of recurrence-free survival in HCC patients (Supplementary Table S4). In the ICGC dataset, the EMT risk score (*p* = 0.002) was an independent predictor of overall survival in HCC patients (Supplementary Table S5).

### Meta-Analysis of Epithelial-Mesenchymal Transition Risk Score With Overall Survival/Recurrence-Free Survival

In the present study, 3 OS and 2 RFS datasets were analyzed. We integrated the hazard ratio in the multivariate Cox regression analysis of the EMT risk score in the OS and RFS datasets. The results of the meta-analysis indicated that a high EMT risk score was a risk factor for overall survival (HR: 2.57; 95% CI: 1.94–3.41, *p* < 0.001) and recurrence-free survival (HR: 1.47; 95% CI: 1.15–1.88, *p* = 0.002) of HCC patients (Supplementary Figure S2).

### Nomogram Construction and Validation

**FIGURE 7:**
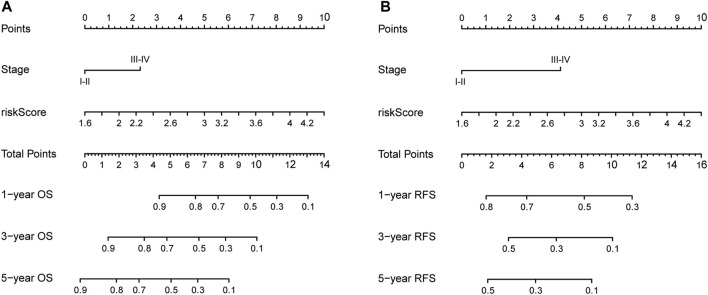
The construction of OS **(A)** and RFS **(B)** predictive nomogram for HCC patients. Notes: OS: overall survival; RFS: recurrence-free survival.

**FIGURE 8:**
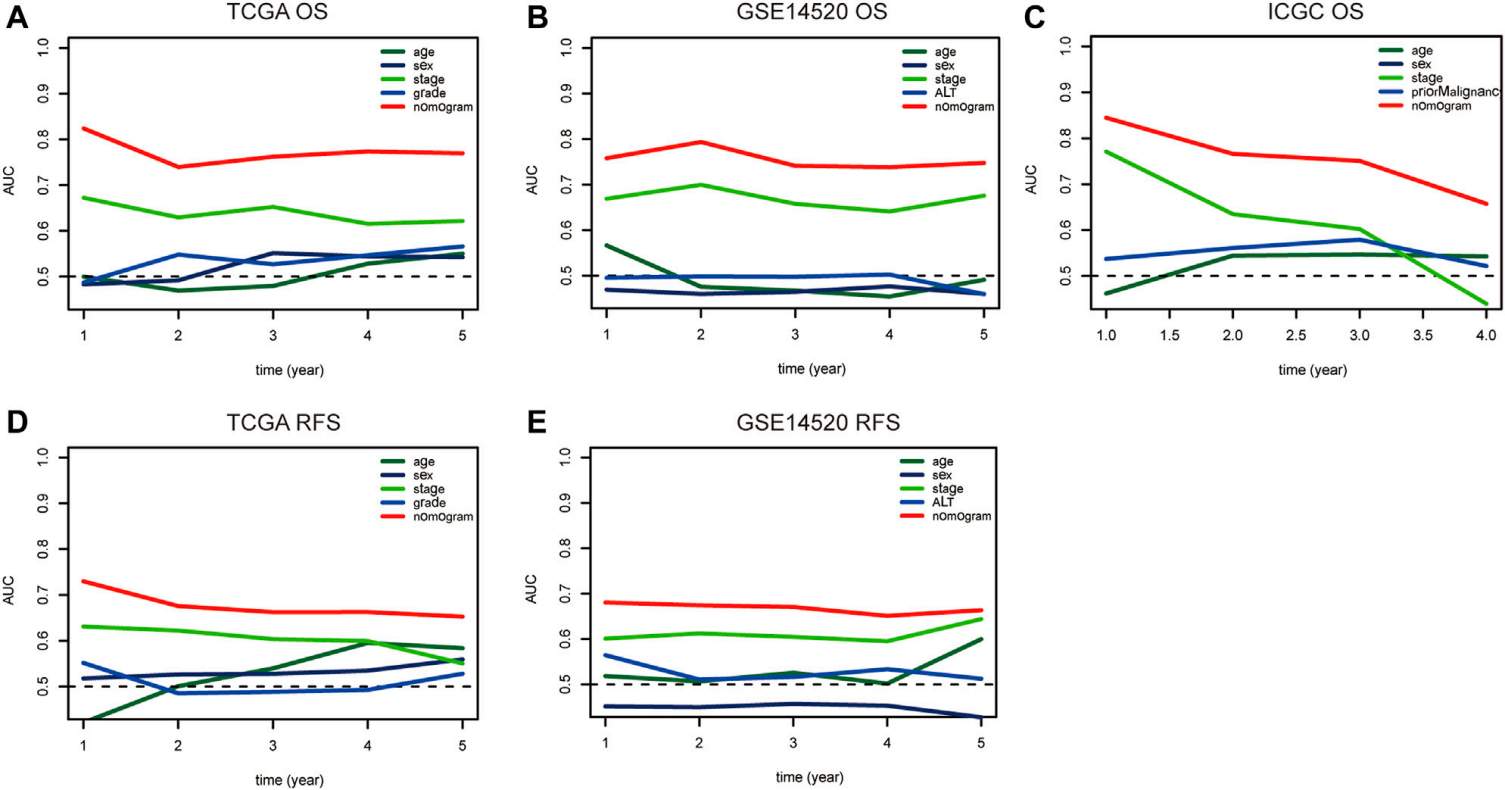
The time-dependent ROC curve is used to evaluate the predictive value between the nomogram model and clinicopathological characteristics in TCGA **(A–D)**, GSE14520 **(B–E)**, and ICGC **(C)** dataset. Notes: ROC: Receiver Operating Characteristic; TCGA: The Cancer Genome Atlas Program; ICGC: International Cancer Genome Consortium.

**FIGURE 9:**
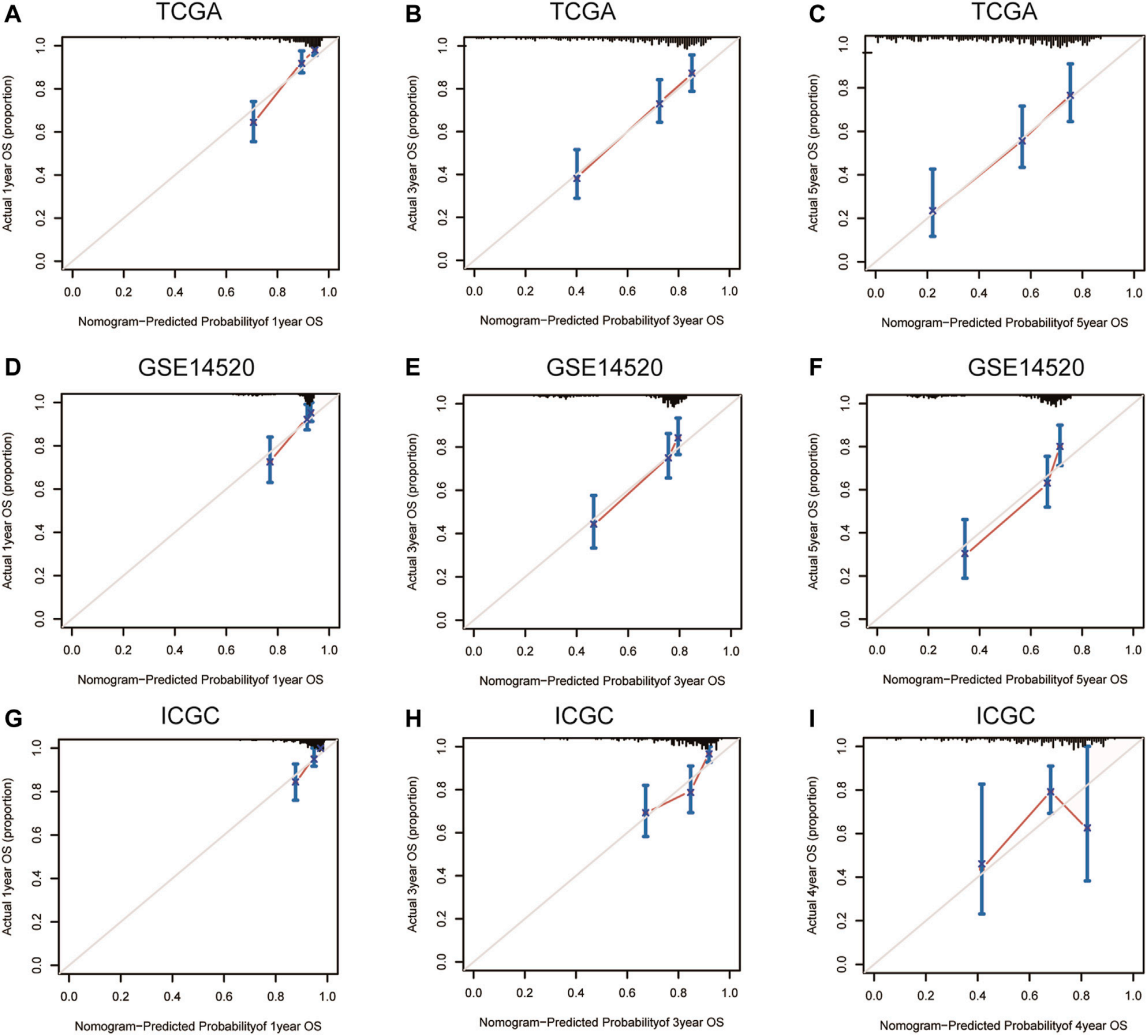
1-year, 3-year, and 5-year calibration curves of the nomogram model in TCGA **(A–C)**, GSE14520 **(D–F)**, and ICGC **(G–I)** OS dataset. Notes: TCGA, The Cancer Genome Atlas Program; ICGC, International Cancer Genome Consortium; OS, overall survival.

**FIGURE 10:**
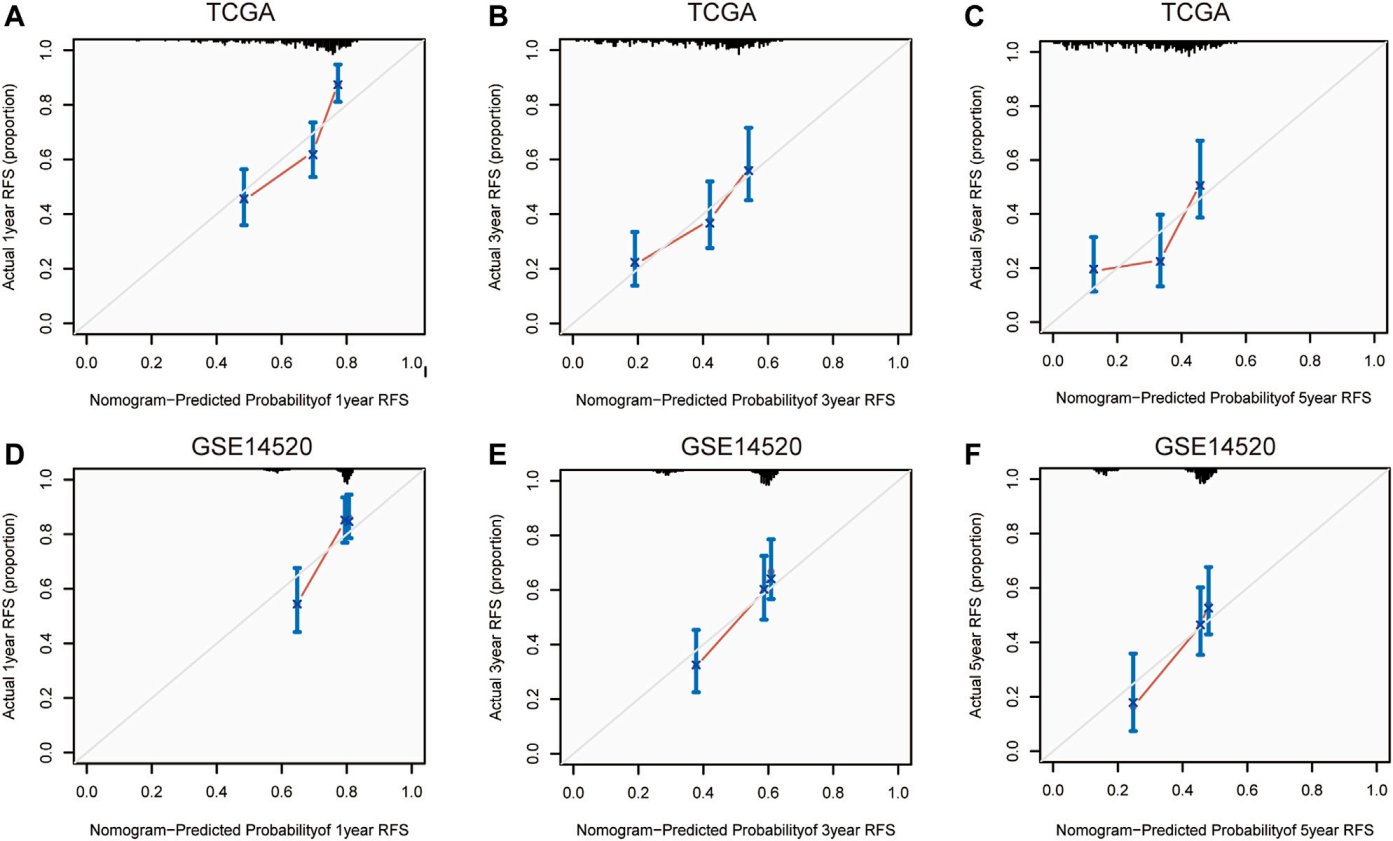
1-year, 3-year, and 5-year calibration curves of the nomogram model in TCGA **(A–C)** and GSE14520 **(D–F)** RFS dataset. Notes: TCGA, The Cancer Genome Atlas Program; ICGC, International Cancer Genome Consortium; RFS, recurrence-free survival.

Nomograms were established to objectively predict the survival outcome (including OS and RFS) of HCC patients at 1, 3, and 5 years. In the TCGA dataset, the EMT risk score and tumor stage were independent risk factors that affected the overall survival and recurrence-free survival of HCC patients. Therefore, OS and RFS nomogram models were constructed based on these two variables (Figure 7). The results of the time-dependent ROC curve showed that the OS and RFS datasets-based nomogram model yielded the best predictive performance compared with other clinicopathological variables (Figure 8). The 1-year, 3-year, and 5-year calibration curves showed that the constructed nomogram model had good accuracy in predicting OS (Figure 9) and RFS (Figure 10).

### Validation of Expression of Selected Epithelial-Mesenchymal Transition Risk Genes

We analyzed the mRNA expression of 9 EMT-related genes in the TCGA, GSE14520, and ICGC datasets and found that these nine genes were dysregulated in these three HCC datasets (Supplementary Figures S3A–C). The HPA database was used to explore the protein expression levels of EMT-related genes. No corresponding data was found for PPARGC1A in the database. The differences in expression of the remaining eight genes between HCC tissue and normal liver tissue is shown in Supplementary Figure S3D. The results showed that HDAC2, HDAC1, SPP1, CKS2, and LGALS3 were significantly increased in HCC tissue compared with normal liver tissue. No significant difference in ENO1, SERPINE1 LMNB1 expression was found between normal liver and HCC tissues.

### Gene Set Enrichment Analysis

**FIGURE 11:**
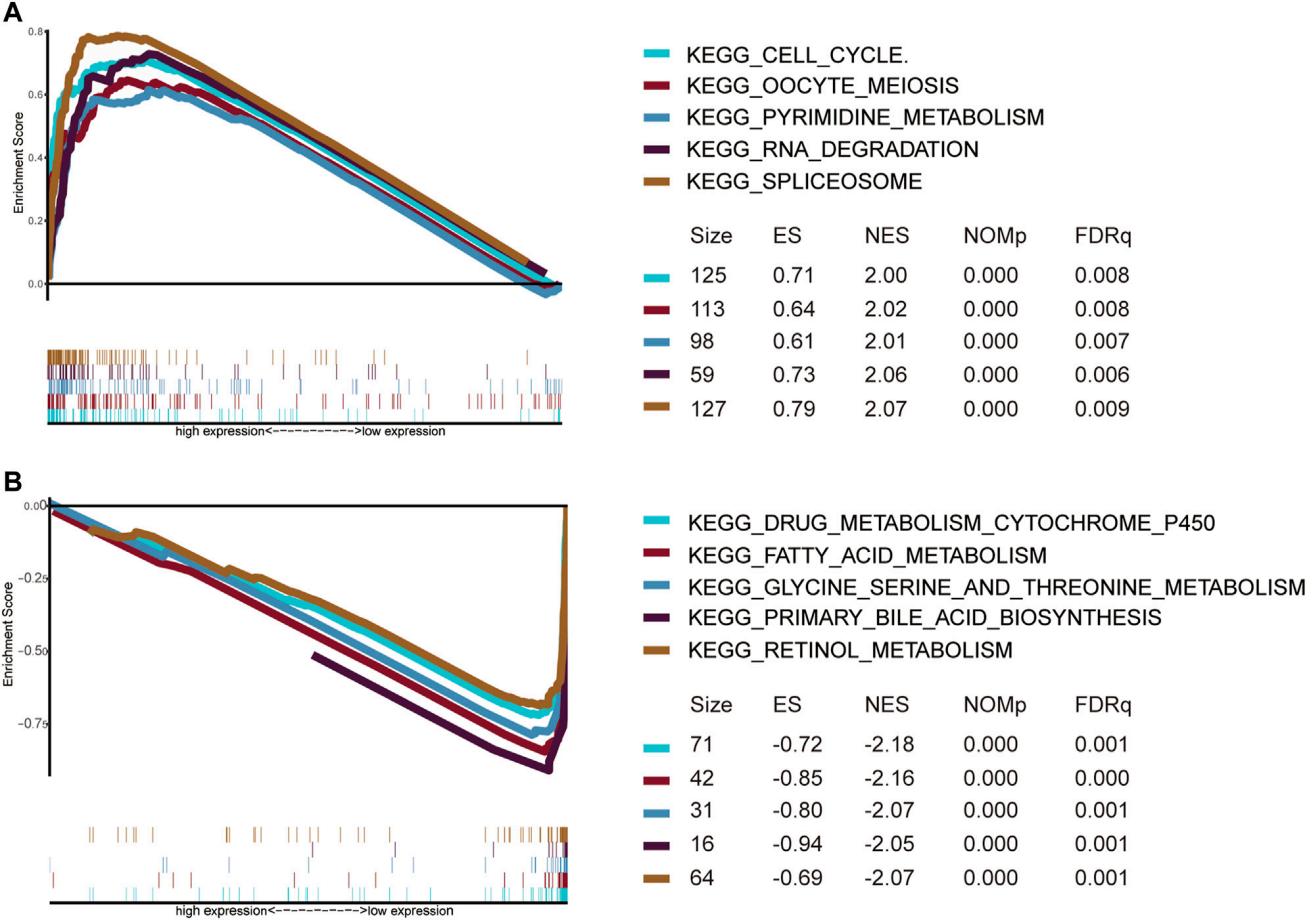
Gene set enrichment analysis of pathways for high **(A)** and low **(B)** EMT risk groups.

GSEA was used to explore significantly enriched KEGG pathways associated with the EMT gene signature in the TCGA dataset. The high EMT risk group was significantly enriched in the spliceosome, RNA degradation, oocyte meiosis, pyrimidine metabolism, and cell cycle (Top 5) (Figure 11A). In contrast, the low EMT risk group was significantly enriched in Drug metabolism cytochrome P450, fatty acid metabolism, Glycine serine and threonine metabolism, retinol metabolism, and Primary bile acid Biosynthesis (Top 5) (Figure 11B).

### Correlation Between the Epithelial-Mesenchymal Transition Risk Score and Immune Infiltration

**FIGURE 12:**
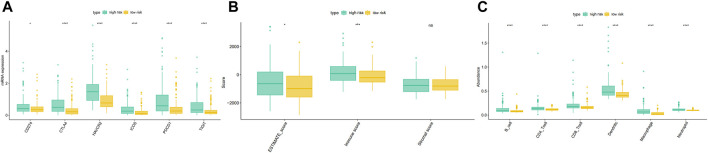
Differences of the immune score, stromal score, ESTIMATE score **(B)**, immune cell **(C)**, and gene expression in immune checkpoints and T cell exhaustion **(A)** between high and low-risk groups in the TCGA dataset.

We downloaded the expression of tumor-infiltrating immune cells, tumor immune score, stroma score, and ESTIMATE score from the TIMER and ESTIMATE databases. Correlation analysis showed a significant correlation between the EMT risk score and six tumor immune cells in the TCGA dataset (Supplementary Figure S4). Moreover, a significant correlation was found between the EMT risk score and immune score (Supplementary Figure S5), further substantiating the relationship between the EMT risk score and immune infiltration levels. Immunotherapy has gained significant momentum in recent years, especially in tumor therapy. Mounting evidence suggests that immunotherapy has significantly prolonged the survival of many cancer patients. In our study, the differences in gene expression in immune checkpoints and T cell exhaustion were analyzed between the high-risk and low-risk groups. The results showed that HCC patients in the high-risk group had higher mRNA expression of immune-related genes (Figure 12A). Moreover, the ESTIMATE score, immune score, B cell, CD4T cell, CD8T cell, Dendritic cell, Macrophage cell, Neutrophil were significantly higher in the high-risk group than in the low-risk group (Figures 12B,C).

## Discussion

HCC is a common malignant tumor well-acknowledged to exert increasing public health and socio-economic burden. Given the limitations of the TNM staging system for predicting prognosis, there is an urgent need for biomarkers to predict the survival of HCC patients. With the rapid development of high-throughput technologies, much emphasis has been placed on gene chip technology since it can be used to identify biomarkers associated with the heterogeneity of HCC (Caruso et al., 2020). Herein, we established an EMT-related risk gene signature to accurately predict the HCC patient prognosis by public database mining.

In recent years, tumor-associated fibroblasts and exosomes have attracted significant attention for their potential role in tumorigenesis. It has been established that cancer-associated fibroblasts (CAFs) are one of the most important cellular components in the tumor stroma and are in a persistently activated state. Indeed, fibroblasts represent an essential and abundant component of the tumor microenvironment, which affects angiogenesis, extracellular matrix remodeling, immunosuppression, and stem cell properties to promote the occurrence and development of HCC (Zhang et al., 2020). Current evidence suggests a negative correlation between CAF expression and HCC patient prognosis (Yin et al., 2019). For instance, Lau et al. found that high α-SMA expression in HCC correlated with shorter disease-free survival than low α-SMA expression (Lau et al., 2016)n. Besides, exosomes are subcellular vesicles 40–130 nm in diameter, exhibiting a spherical or cup-shaped appearance under electron microscopy. Exosomes are widely acknowledged to originate from early intracellular endosomes, which bud inward to form multivesicular endosomes, fusing with the cell membrane to release small vesicles outside the cell to form exosomes. Over the years, exosome-related proteins, miRNAs, lncRNAs, and circRNAs have been extensively studied as prognostic markers for HCC patients (Zhang et al., 2021). For example, Sun et al. demonstrated that S100A4 carried by exosomes in the plasma of HCC patients could promote HCC metastasis, thereby affecting patient OS and DFS (Sun et al., 2021). Compared with our constructed EMT-gene signature, both CAFs and exosomes could be used as prognostic indicators for HCC patients. However, it should be borne in mind that exosomes generally come from body fluids such as plasma, urine, bronchoalveolar lavage fluid, and synovial fluid, while our indicators generally come from RNA sequencing of tumor tissue samples. Moreover, CAFs in HCC tissues are widely believed to come from fibroblasts in surrounding tumor tissue, hepatic sinusoidal endothelial cells, mesenchymal stromal cells, and hepatic stellate cells. Accordingly, circulating exosomes that can target EMT proteins may have huge prospects for application in predicting the prognosis of HCC and providing a new therapeutic target.

Herein, we integrated four HCC microarrays in the GEO dataset through the metaDE package and finally identified 317 differentially expressed EMT-related genes. Then, univariate Cox regression analysis was used to explore the prognostic-related EMT genes in the TCGA and GSE14520 datasets and the Lasso Cox regression model to identify the EMT gene signature. Next, we validated the diagnostic and prognostic value of the EMT-related gene signature for predicting survival outcomes in the three HCC datasets and constructed a predictive nomogram of OS and RFS based on the EMT risk score and tumor stage. The constructed nomogram exhibited good consistency during prediction in the training and validation datasets. A high EMT risk score correlated with poor prognosis, high TNM stage, poor histopathological grade, and immune infiltration in the HCC dataset.

Among the screened EMT-related genes, some have already been extensively studied in liver cancer, especially HDAC2. In this regard, Ji Heon Noh et al. found that HDAC2 was upregulated in liver cancer tissues and could promote the proliferation of HCC cells through the expression of G1/S cyclin (Noh et al., 2011). Yang et al. found that HDAC2 overexpression could increase HCC cell mobility and doxorubicin resistance (Yang et al., 2019). Besides, Zhao et al. found that liver cancer could promote metastasis through the HDAC1/FAM99A/miR-92a axis under a hypoxic environment (Zhao et al., 2020). Moreover, Wang et al. found that silencing SPP1 could inhibit liver cancer cell proliferation and promote cell apoptosis, which miR-181c regulated (Wang et al., 2019). Ji et al. found that CKS2 could encourage the expansion of HCC cells by downregulating PTEN (Ji et al., 2018). Song et al. reported that LGALS3 promoted the occurrence and metastasis of liver cancer through the β-catenin signaling pathway (Song et al., 2020). Moreover, Jiang et al. documented that ENO1 secreted by exosomes could regulate the expression of integrin α6β4 and promote liver cancer progression (Jiang et al., 2020). Divella et al. found that SERPINE1 polymorphism can be used as a prognostic indicator for HCC patients receiving TACE, but its underlying molecular mechanism has not been explored (Divella et al., 2015). Finally, Sun et al. reported that serum LMNB1 levels could be used as a diagnostic marker for early HCC, with high sensitivity and specificity. However, its functions in HCC remain poorly understood (Sun et al., 2010). No study has hitherto studied the role of PPARGC1A in liver cancer, warranting further studies.

Over the years, the advent of high-throughput technology has resulted in the emergence of multi-omics integrated analysis. Nowadays, researchers can obtain large-scale omics data from distinct molecular levels, including genome, transcriptome, proteome, interactome, epigenome, metabolome, liposome, and microbiome data, to conduct in-depth disease research. Interestingly, an omics analytical method can provide information on biological processes that differ in different life processes or disease groups. However, these analyses often have limitations. Multi-omics approaches are well-established to integrate information from several omics levels to provide evidence to better understand biological mechanisms and candidate key factors, leading to a deeper understanding of the molecular mechanisms and genetic basis of complex traits in biological processes and disease processes (Song et al., 2015; Su et al., 2020). In the present study, we substantiated the potential role of the screened EMT-related genes through multi-omics analysis. For instance, HDAC2 was identified as an EMT-related gene screened in our research. It is well-established that the TCGA database can be used to analyze changes in mRNA expression, protein level, methylation level, and DNA copy number in HCC tissues to explore potential factors underlying increased HDAC2 expression in HCC tissue. Finally, to further explore the possible mechanisms underlying its functions, correlation analysis can be performed to determine its co-expressed genes and GSEA to determine the functions of HDAC2 in HCC. Therefore, this multi-omics approach provides novel insights that provide the foothold to explore HCC tumorigenesis mechanisms.

Our study identified a nine-gene signature to predict the overall survival and recurrence-free survival of HCC patients. It yielded good prediction accuracy, providing potential targets for individualized treatment of HCC. At the same time, we acknowledge there were limitations and shortcomings in this study. In this regard, the molecular mechanism of the selected EMT-related proteins in hepatocellular carcinoma was not explored. The relatively small sample size of our study population highlights the need for a large and multiple HCC cohort to validate the established gene signature and increase the robustness of our findings.

## Data Availability

The datasets presented in this study can be found in online repositories. The names of the repository/repositories and accession number(s) can be found in the article/Supplementary Material.
