# Supplementary material for: A Novel Epithelial-Mesenchymal Transition Gene Signature Correlated With Prognosis, and Immune Infiltration in Hepatocellular Carcinoma
Source: Front Pharmacol. 2022 Apr 20;13:863750. doi: 10.3389/fphar.2022.863750 (PMC9065556; doi:10.3389/fphar.2022.863750)
Supplement: Supplementary file 1 [file Table1.DOCX]

**Supplementary table1**. Univariate and multivariate regression analysis of risk factors affecting OS in the TCGA dataset.

| Variable | Univariate analysis | | | Multivariate analysis | | |
| --- | --- | --- | --- | --- | --- | --- |
|  | P-value | HR | 95%CI | P-value | HR | 95%CI |
| Age (≥60 / <60) | 0.414 | 1.172 | 0.800-1.717 |  |  |  |
| Sex (female / male) | 0.162 | 1.320 | 0.894-1.948 |  |  |  |
| TNM stage (IV+III/II+I) | **<0.001** | **2.835** | **1.934-4.157** | **<0.001** | **2.456** | **1.666-3.622** |
| Histologic grade (G4+G3/G2+G1) | 0.660 | 1.091 | 0.739-1.611 |  |  |  |
| Risk (high/low) | **<0.001** | **2.940** | **1.954-4.422** | **<0.001** | **2.598** | **1.719-3.928** |

Notes: OS: overall survival; TCGA: The Cancer Genome Atlas; TNM: Tumor Node Metastases; HR: hazard ratio; 95%CI: 95%confidence interval.

**Supplementary table2**. Univariate and multivariate regression analysis of risk factors affecting RFS in the TCGA dataset.

| Variable | Univariate analysis | | | Multivariate analysis | | |
| --- | --- | --- | --- | --- | --- | --- |
|  | P-value | HR | 95%CI | P-value | HR | 95%CI |
| Age (≥60 / <60) | 0.803 | 0.961 | 0.703-1.314 |  |  |  |
| Sex (female / male) | 0.314 | 1.187 | 0.850-1.658 |  |  |  |
| TNM stage (IV+III/II+I) | **<0.001** | **1.728** | **1.448-2.061** | **<0.001** | **1.630** | **1.360-1.953** |
| Histologic grade (G4+G3/G2+G1) | 0.553 | 1.101 | 0.800-1.515 |  |  |  |
| Risk (high/low) | **<0.001** | **1.906** | **1.390-2.613** | **0.005** | **1.595** | **1.153-2.208** |

Notes: RFS: recurrence-free survival; TCGA: The Cancer Genome Atlas; TNM: Tumor Node Metastases; HR: hazard ratio; 95%CI: 95%confidence interval.

**Supplementary table3**. Univariate and multivariate regression analysis of risk factors affecting OS in the GSE14520 dataset.

| Variable | Univariate analysis | | | Multivariate analysis | | |
| --- | --- | --- | --- | --- | --- | --- |
|  | P-value | HR | 95%CI | P-value | HR | 95%CI |
| Age (≥60 / <60) | 0.449 | 0.801 | 0.451-1.423 |  |  |  |
| Sex (female / male) | 0.156 | 0.590 | 0.285-1.224 |  |  |  |
| ALT (>50/50) | 0.751 | 1.073 | 0.696-1.653 |  |  |  |
| TNM stage (IV+III/II+I) | **<0.001** | **3.513** | **2.240-5.511** | **<0.001** | **2.909** | **1.836-4.610** |
| Risk (high/low) | **<0.001** | **2.820** | **1.788-4.448** | **<0.001** | **2.378** | **1.492-3.790** |

Notes: OS: overall survival; ALT: alanine transaminase; TNM: Tumor Node Metastases; HR: hazard ratio; 95%CI: 95%confidence interval.

**Supplementary table4**. Univariate and multivariate regression analysis of risk factors affecting RFS in the GSE14520 dataset.

| Variable | Univariate analysis | | | Multivariate analysis | | |
| --- | --- | --- | --- | --- | --- | --- |
|  | P-value | HR | 95%CI | P-value | HR | 95%CI |
| Age (≥60 / <60) | 0.845 | 0.956 | 0.606-1.506 |  |  |  |
| Sex (female / male) | **0.019** | **0.461** | **0.241-0.881** | 0.050 | 0.521 | 0.271-1.001 |
| ALT (>50/50) | 0.224 | 1.250 | 0.872-1.791 |  |  |  |
| TNM stage (IV+III/II+I) | **<0.001** | **2.260** | **1.514-3.373** | **0.001** | **1.987** | **1.312-3.007** |
| Risk (high/low) | **0.019** | **1.537** | **1.072-2.203** | 0.140 | 1.323 | 0.913-1.919 |

Notes: RFS: recurrence-free survival; ALT: alanine transaminase; TNM: Tumor Node Metastases; HR: hazard ratio; 95%CI: 95%confidence interval.

**Supplementary table5** Univariate and multivariate regression analysis of risk factors affecting OS in the ICGC dataset.

| Variable | Univariate analysis | | | Multivariate analysis | | |
| --- | --- | --- | --- | --- | --- | --- |
|  | P-value | HR | 95%CI | P-value | HR | 95%CI |
| Age (≥60 / <60) | 0.483 | 0.774 | 0.379-1.581 |  |  |  |
| Sex (female / male) | **0.022** | **0.477** | **0.253-0.897** | **-** |  |  |
| TNM stage (IV+III/II+I) | **0.012** | **2.203** | **1.191-4.076** | 0.053 | 1.846 | 0.991-3.439 |
| Prior-Malignancy (yes/no) | 0.131 | 1.885 | 0.829-4.289 |  |  |  |
| Risk (high/low) | **0.001** | **3.282** | **1.641-6.565** | **0.002** | **2.973** | **1.470-6.011** |

Notes: OS: overall survival; ICGC: International Cancer Genome Consortium; TNM: Tumor Node Metastases; HR: hazard ratio; 95%CI: 95%confidence interval.
