# Supplementary figures and images for: A Novel Epithelial-Mesenchymal Transition Gene Signature Correlated With Prognosis, and Immune Infiltration in Hepatocellular Carcinoma
Source: Front Pharmacol. 2022 Apr 20;13:863750. doi: 10.3389/fphar.2022.863750 (PMC9065556; doi:10.3389/fphar.2022.863750)

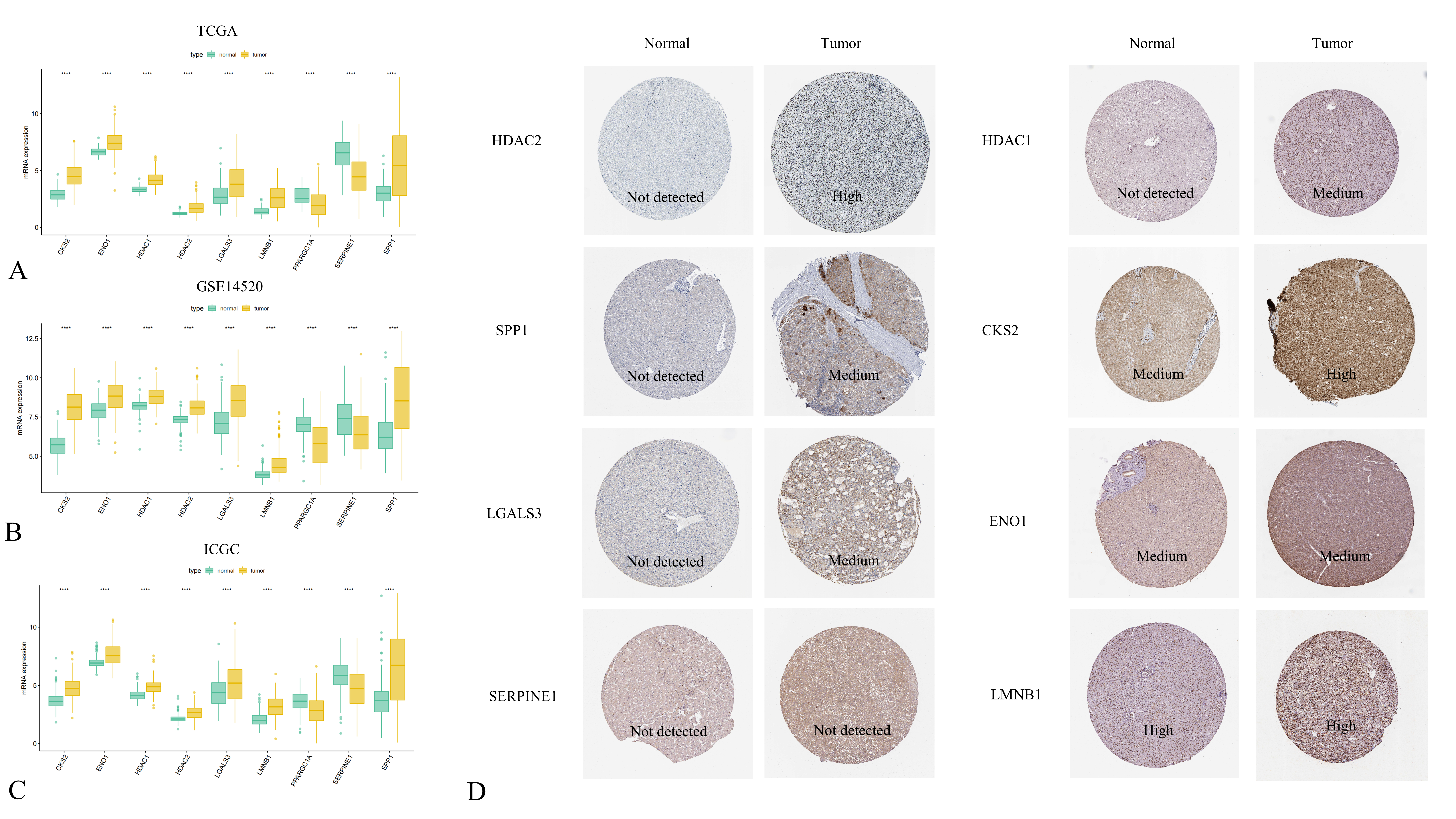

Supplement: Supplementary file 2 [file Image3.TIF]

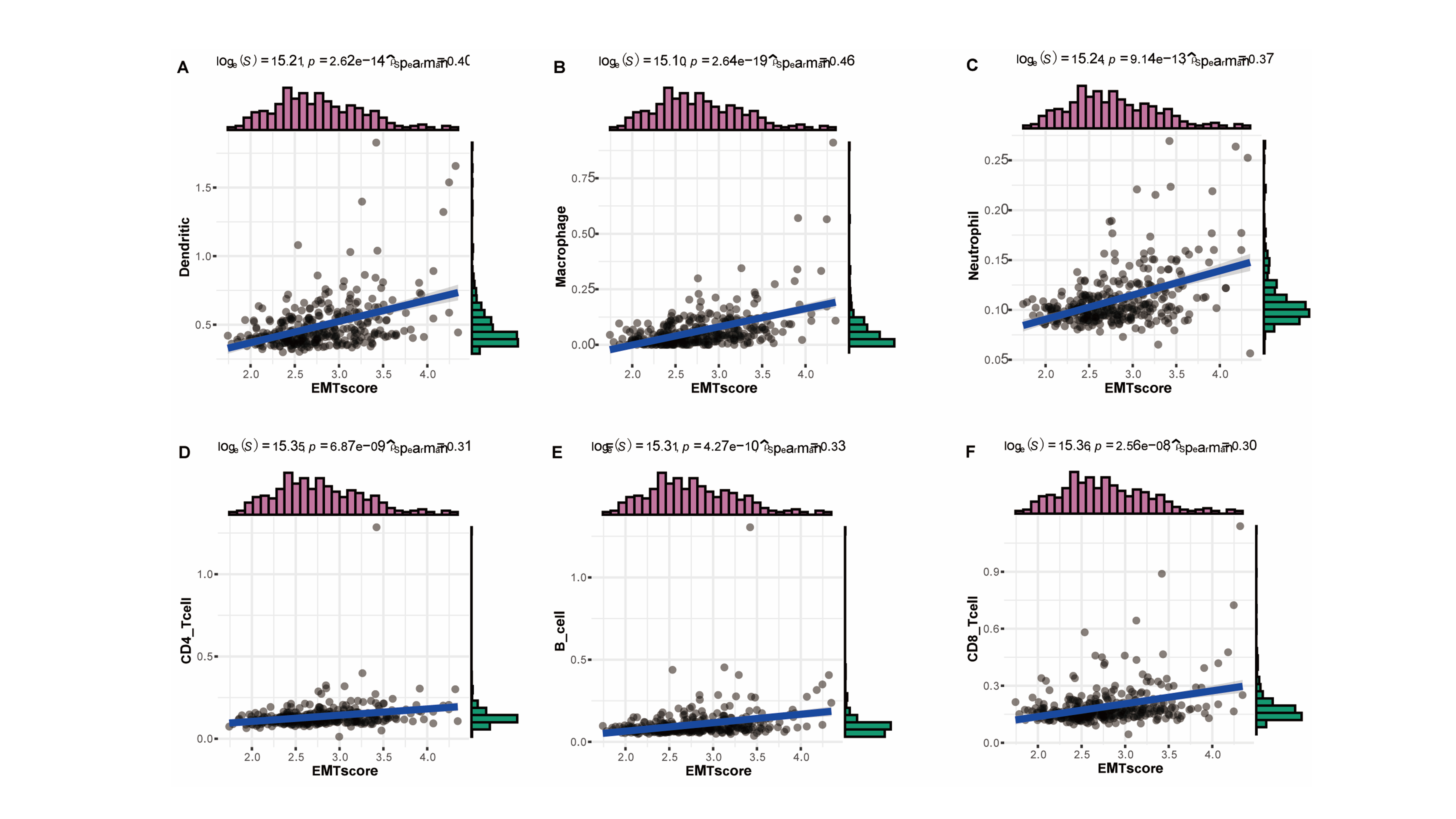

Supplement: Supplementary file 3 [file Image4.TIF]

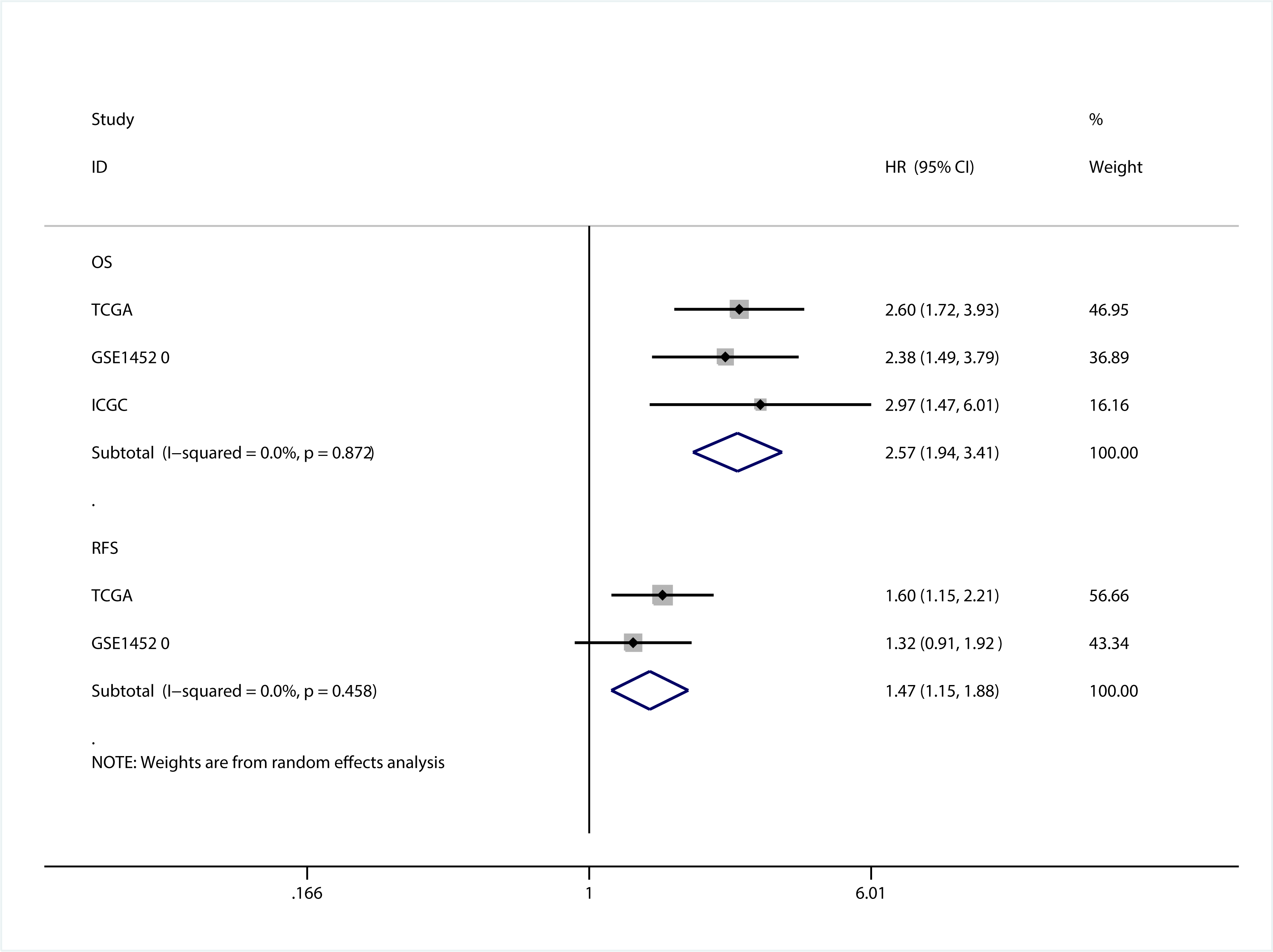

Supplement: Supplementary file 4 [file Image2.TIF]

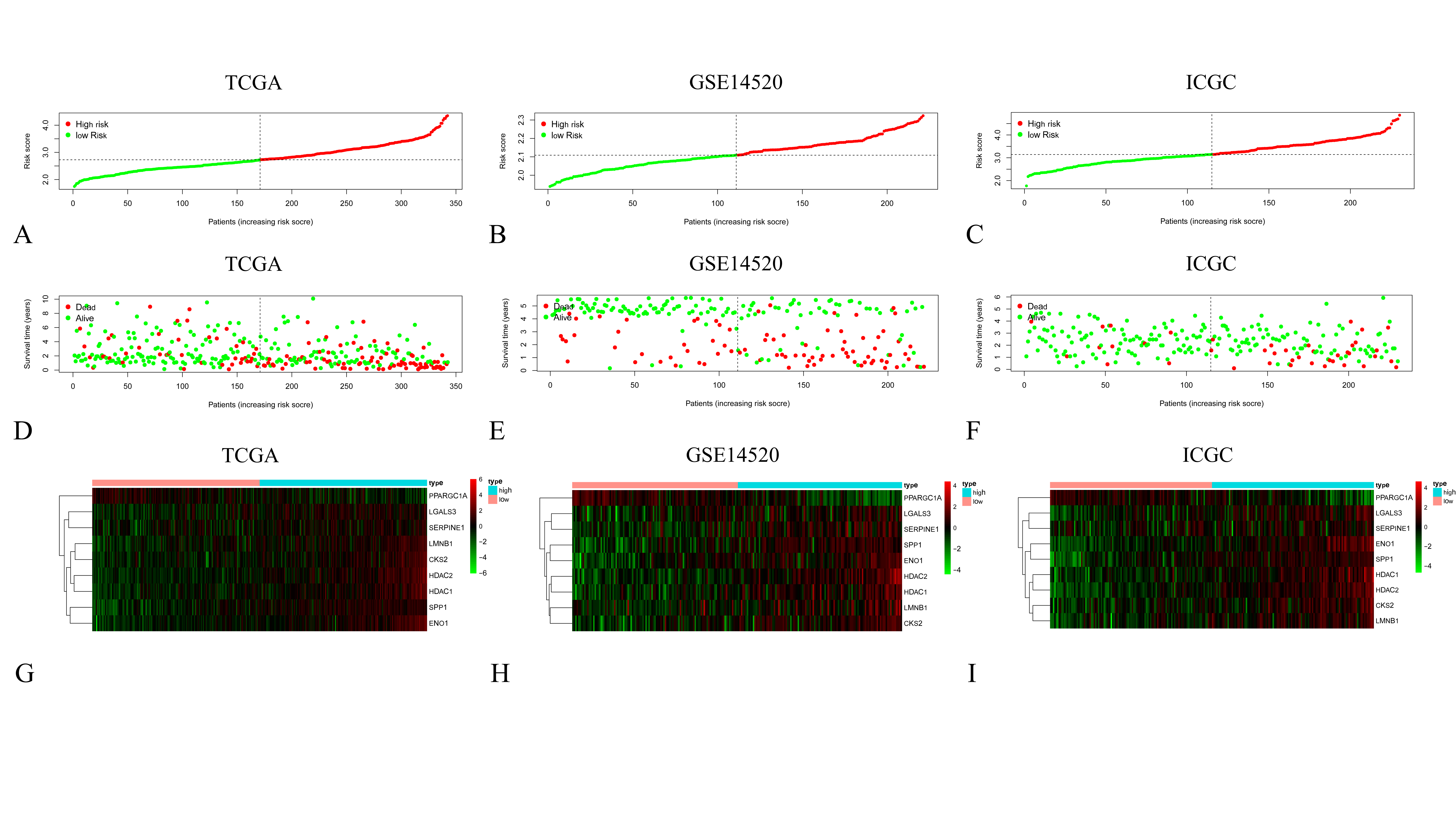

Supplement: Supplementary file 5 [file Image1.TIF]

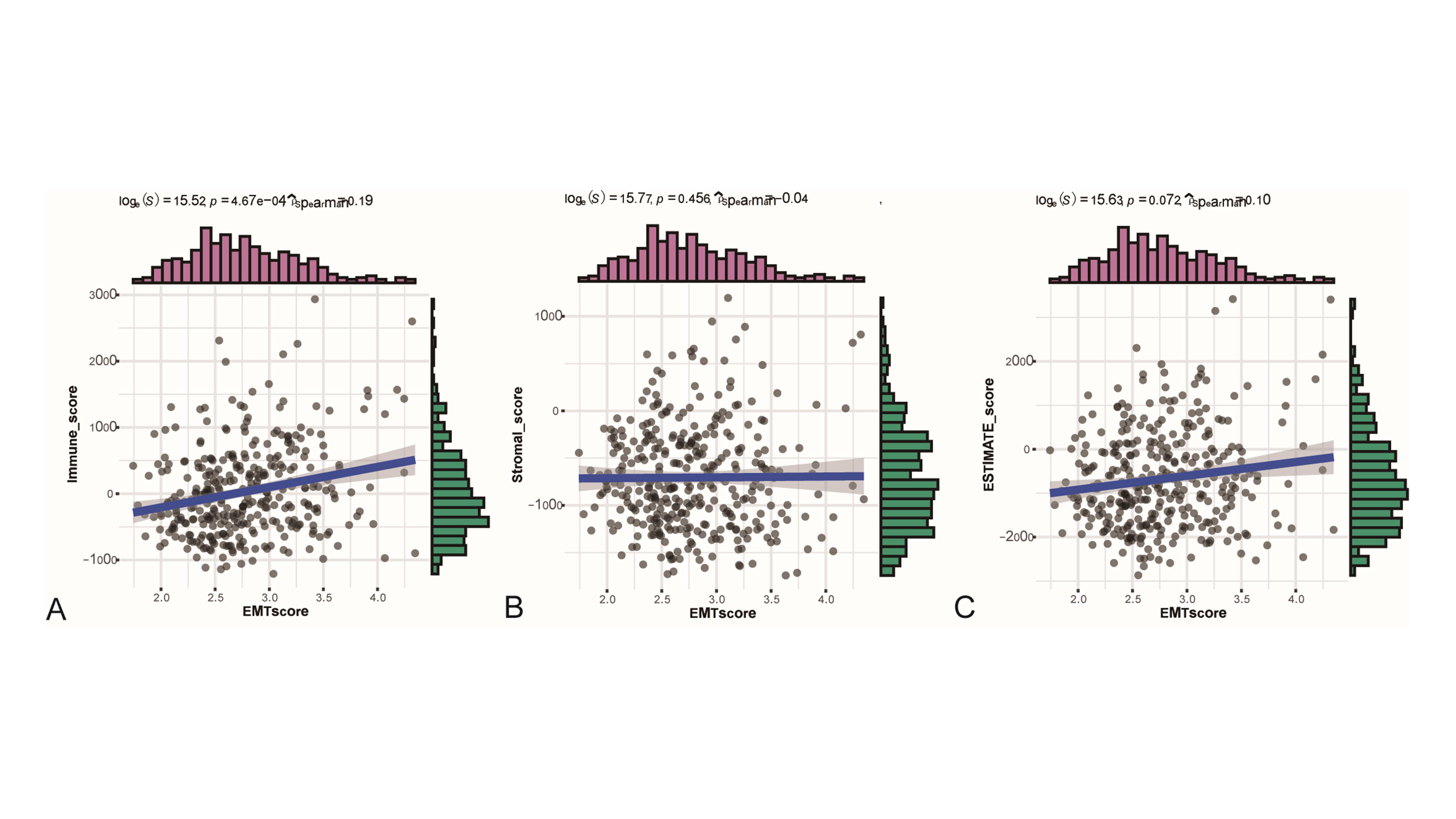

Supplement: Supplementary file 6 [file Image5.TIF]
